# Exploring neuromarketing’s influence on consumer impulsivity through the lens of personality traits

**DOI:** 10.3389/fpsyg.2026.1778248

**Published:** 2026-03-18

**Authors:** Kanika Nagpal, Kiran Bala, Sumanjeet Singh, Arun Yadav, Minakshi Paliwal

**Affiliations:** 1 Department of Commerce, Delhi School of Economics, University of Delhi, New Delhi, India; 2 Department of Commerce, Ramjas College, University of Delhi, New Delhi, India; 3 Department of Marketing and Supply Chain Management, Central University of Jammu, Jammu, India; 4 Department of Commerce, Daulat Ram College, University of Delhi, New Delhi, India

**Keywords:** cognitive processing, consumer impulsivity, consumer traits, emotional appeals, neuromarketing, neuro-pricing strategies

## Abstract

**Background:**

This study examines the effectiveness of neuromarketing stimuli shaping consumer impulsive behaviour and investigates the moderating role of consumer traits. Addressing a key gap in the literature, it integrates the Stimulus–Organism–Response (S–O–R) framework with dual-process theory to explain how neuromarketing stimuli interact with individual dispositional characteristics to influence impulsive buying behaviour.

**Method:**

A quantitative research design was employed using survey data from 609 digitally active consumers from Delhi–NCR region, India, who frequently interacted with neuromarketing-driven content. Partial least squares structural equation modelling (PLS-SEM) was applied to assess relationships among six neuromarketing determinants: emotional appeals, scarcity and urgency cues, sensory triggers, endorsement influence, neuro-pricing strategies, and cognitive processing, and their effects on neuromarketing efficacy and consumer impulsivity.

**Findings:**

Emotional appeals, sensory triggers, neuro-pricing strategies, and cognitive processing significantly enhanced perceived neuromarketing efficacy, which in turn was positively associated with consumer impulsivity. In contrast, scarcity and urgency cues as well as endorsement influence did not demonstrate significant effects. Consumer traits exerted a direct influence on impulsive behaviour and strengthened the positive relationship between neuromarketing efficacy and consumer impulsivity.

**Conclusion:**

The findings indicate that neuromarketing effectiveness is mediated by both affective and cognitively reinforced mechanisms and conditioned by dispositional characteristics. By incorporating trait-based boundary conditions within a unified structural framework, this research advances theoretical understanding of impulsive consumption in digitally amplified environments and provides evidence-based guidance for responsible marketing practice.

## 1 Introduction

Consumers are exposed to an unprecedented volume of marketing stimuli across digital platforms. In such saturated environments, only a limited number of messages successfully capture attention and influence decision-making (Alsharif et al., 2024). Growing evidence indicates that consumer responses to marketing communications vary, individuals process information differently, i.e., some are primarily emotionally driven, others rely on structured cognitive evaluation, and some respond to sensory cues embedded within marketing content (Lim, 2018). Understanding these psychological differences has therefore become central to contemporary consumer research.

Neuromarketing has emerged as an interdisciplinary domain integrating neuroscience and consumer psychology to investigate subconscious drivers of behaviour (Bhardwaj et al., 2023). Through tools such as functional magnetic resonance imaging (fMRI), electroencephalography (EEG), and eye-tracking, researchers have examined activation of emotional and attentional systems through marketing stimuli (Aldayel et al., 2021). The following approaches have substantially deepened understanding of neural and cognitive reactions to advertisements.

Despite increasing scholarly attention, important theoretical gaps exist. Traditional marketing models largely assume that consumer decisions are rational and intention-based. In contrast, behavioural research demonstrates that many purchase decisions are spontaneous, emotionally activated, and heuristic-driven (Kakaria et al., 2023). Although prior studies confirm that neuromarketing stimuli, such as emotional appeals, sensory cues, and pricing heuristics can influence purchase behaviour, limited empirical work posit variability in consumer responsiveness. Specifically, existing literature offers limited insights into why some consumers translate neuromarketing-induced impulses into actual purchases, whereas others resist such influence.

Emerging research proposes that stable consumer traits shape how marketing information is interpreted, evaluated, and enacted (Sharma et al., 2010). However, factual integration of dispositional characteristics within neuromarketing frameworks remains underdeveloped. Most studies emphasize direct stimulus–response mechanisms or laboratory-based neural activation, often overlooking personality-driven boundary conditions that may amplify or attenuate marketing effectiveness in real-world contexts.

To address this gap, the present study adopts the Stimulus–Organism–Response (S–O–R) framework to examine the psychological mechanisms underlying impulsive consumption in digitally driven environments. While neuromarketing research often employs neurophysiologic tools such as electroencephalography (EEG) or functional magnetic resonance imaging (fMRI) to capture neural responses, the present study adopts a perception-based approach to examine how consumers respond to marketing stimuli informed by neuromarketing principles. Within this framework, neuromarketing determinants are conceptualized as stimuli, neuromarketing efficacy as the organismic evaluative mechanism reflecting consumers’ perceived effectiveness of such stimuli, and consumer impulsivity as the behavioural response. Consumer traits are introduced as moderating variables to capture dispositional heterogeneity in responsiveness to neuromarketing practices.

Accordingly, this study aims to (1) identify key neuromarketing determinants influencing perceived neuromarketing efficacy, (2) examine the mediating role of neuromarketing efficacy in driving consumer impulsive behaviour, and (3) investigate whether consumer traits moderate the relationship between neuromarketing efficacy and impulsivity. By integrating affective, cognitive, and trait-based dimensions within a unified structural model tested on a large consumer sample, this research advances theoretical understanding of impulsive consumption in neuromarketing contexts. It contributes to psychology-oriented consumer research by demonstrating that neuromarketing effectiveness is both mechanism-driven and trait-contingent rather than universally uniform.

## 2 Underpinning theory and hypotheses development

### 2.1 Theoretical foundation

Neuromarketing has reshaped contemporary understanding of consumer decision-making by integrating insights from neuroscience and psychology into marketing strategy (Garczarek-Bąk et al., 2021). It enables systematic examination of how marketing stimuli activate affective and cognitive processes that ultimately shape behavioural outcomes. However, existing research has frequently examined neuromarketing elements in isolation, with limited integration of stimulus characteristics, internal evaluative mechanisms, and individual-level heterogeneity within a unified theoretical model.

The present study is grounded in the S–O–R framework (Mehrabian and Russell, 1974). Within this framework, Stimuli (S) represents external environmental cues, Organism (O) reflects internal psychological and evaluative processes, and Response (R) denotes observable behavioural outcomes. In neuromarketing contexts, external advertising cues influence internal evaluative states, which subsequently shape purchasing behaviour.

In the current model, emotional appeals, scarcity and urgency cues, sensory triggers, neuro-pricing strategies, endorsement influence, and cognitive processing cues are conceptualized as stimulus components. These stimuli are hypothesized to influence internal evaluative processes, operationalised as neuromarketing efficacy, which is elucidated as consumer’s perceived psychological effectiveness of neuromarketing-based stimuli in influencing their cognitive and affective evaluations. This organismic assessment subsequently predicts consumer impulsive behaviour, defined as spontaneous and unplanned purchasing actions driven by immediate psychological activation.

To further clarify the organismic mechanism, this study incorporates Dual-Process Theory (Evans and Stanovich, 2013), which distinguishes between intuitive, affect-driven processing (System 1) and analytical, deliberative processing (System 2). Neuromarketing stimuli frequently activate rapid evaluative processes associated with System 1; however, structured informational cues may also reinforce credibility through System 2 processing. Integrating these perspectives provides a more comprehensive explanation of how neuromarketing cues influence perceived efficacy and subsequent impulsive responses.

**Figure 1:**
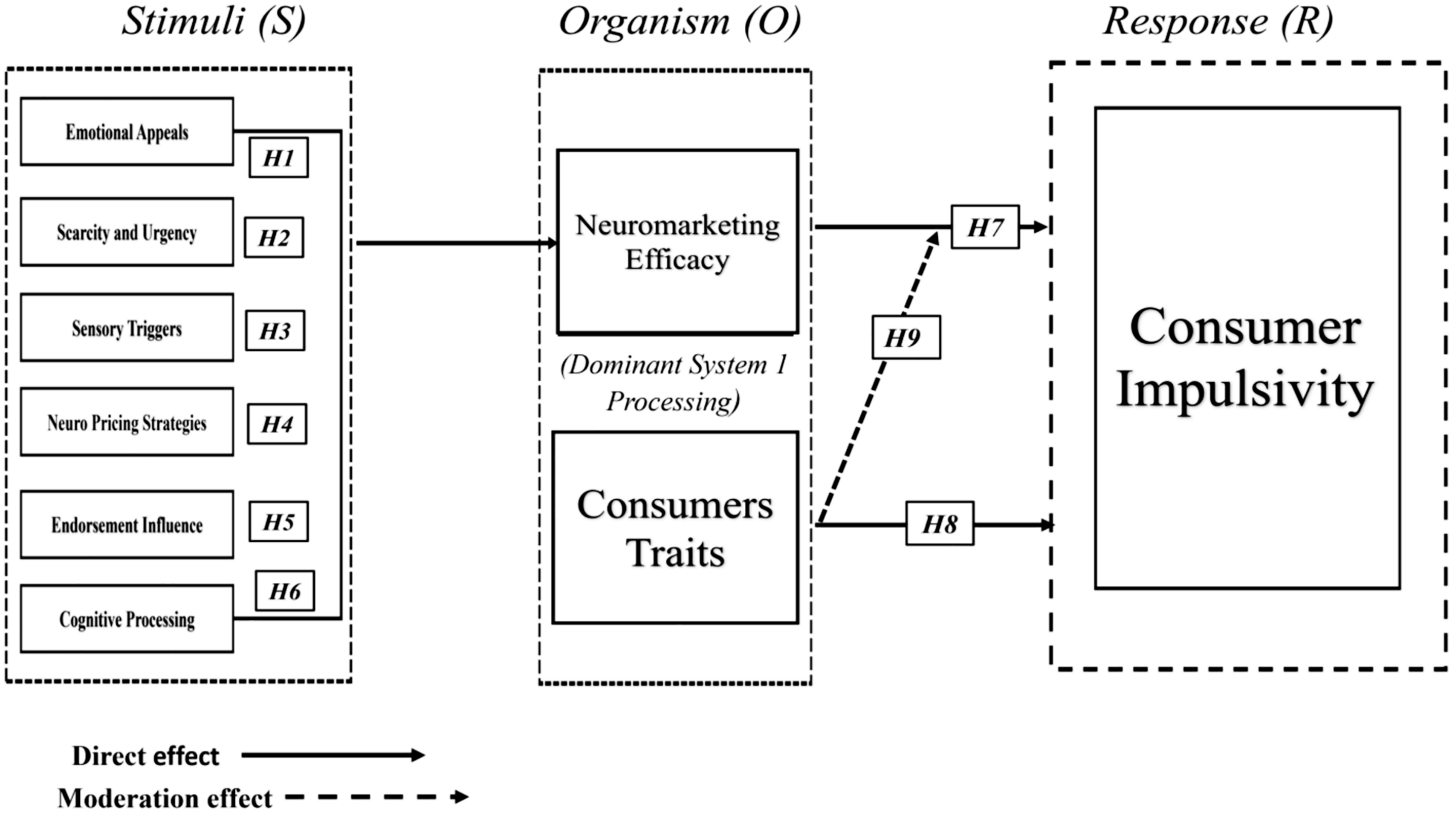
Conceptual framework.

Importantly, consumer traits are introduced as moderating variables. Consumer traits are defined as relatively stable intrinsic characteristics that influence individual’s perception, interpretation, and response to persuasive marketing stimuli. Drawing on personality and consumer behaviour research (Sharma et al., 2010), the present study focuses on traits reflecting emotional susceptibility, fear of missing out (FOMO), and social influence sensitivity. Incorporating these traits enables the model to account for heterogeneity in responsiveness to neuromarketing practices and positions dispositional characteristics as boundary conditions within the S–O–R framework. Together these constructs establish a robust conceptual model (Figure 1) grounded in S–O–R framework.

### 2.2 Neuromarketing determinants as stimuli

#### 2.2.1 Emotional appeals

Emotional appeals refer to advertising strategies designed to evoke affective responses such as excitement, pleasure, aspiration, or fear (Zhang et al., 2014). Accordingly, emotional appeals are expected to positively influence neuromarketing efficacy. Neuroscientific evidence suggests that emotionally salient stimuli enhance memory encoding and reduce cognitive resistance (Vences et al., 2020). Emotionally driven advertising messaging such as invoking happiness, fear, or nostalgia, are more likely to influence consumer attitudes and behaviours. Emotional stimulation facilitates intuitive evaluation and increases perceived persuasive strength (Rodríguez-Hernández et al., 2024) as human’s decision making is inherently guided emotional processes. Studies emphasize that the deeper psychological connections forged through emotionally salient marketing stimuli establish subconscious bonds with customers and foster trust, which transcends mere rational evaluation (Poels and Dewitte, 2019). For example, advertisement storytelling that features children often creates irresistible connections by evoking emotions of joy, care or purity. Previous neuromarketing research supports the notion by quantifying the neural responses to emotional marketing appeals using tools like EEG and fMRI (Golnar-Nik et al., 2019). These responses are associated with instant gratification and limited self-control (Zito et al., 2021), both of which are known predictors of impulsive behaviour (Bak et al., 2022). Within framework of neuromarketing, our study seeks to evaluate the influence of emotional stimuli as a catalyst in driving impulsive buying behaviour among consumers. The following hypothesis is proposed based on the preceding arguments:

*H1*: Emotional appeals significantly impact neuromarketing efficacy in advertisement.

#### 2.2.2 Scarcity and urgency cues

Scarcity and urgency cues emphasize limited availability or time-bound opportunities (Badgaiyan and Verma, 2015). These strategies are intended to activate perceived loss aversion and FOMO, thereby intensifying motivational pressure. By highlighting exclusivity or time sensitivity, such cues may enhance perceived persuasive intensity (Barton et al., 2022). The strategic use of scarcity and urgency cues in advertising harnessing consumer psychology by highlighting limited availability and the imminent expiration of an opportunity (He and Oppewal, 2018). However, its effectiveness may depend on perceived authenticity and consumer trust. The power of these strategies has been long leveraged by marketers in making the product desirable and stimulating immediate consumer action (Yao and Wang, 2024). Such strategy induces action by augmenting the perceived value, rarity and immediacy that is hard to resist (Hamilton and Shaheen Hosany, 2023), exemplified by the allure of Amazon’s lightening deals, the ticking clock or messages “only a few left in stock-order soon.” The key factors in decision-making process are rooted in the principle of FOMO (Zito et al., 2021). In the realm of neuromarketing, this phenomenon has been substantiated by various studies employing neuro-imaging techniques to demonstrate the desirability at the neural level, particularly in relation reward anticipation, value computation and risk assessment (Casado-Aranda et al., 2023). This heightened emotional state can potentially override deliberative decision-making process, resulting in increased susceptibility to impulsive buying. However, the current body of literature has examined these factors in isolation, lacking an integrative analysis of scarcity-based tactics within a neuromarketing context and how differences in individual traits moderate this relationship. Hence, this research extends the inquiry by proposing following hypothesis:

*H2*: Scarcity and urgency cues significantly impact neuromarketing efficacy in advertisement.

#### 2.2.3 Sensory triggers

Sensory triggers involve the strategic engagement of sight, auditory, taste, or other sensory elements to enhance experiential immersion (Aydınoğlu and Sayın, 2016). Multisensory integration strengthens affective engagement and subconscious processing (Marucci et al., 2021), thereby increasing perceived neuromarketing efficacy. Empirical studies show that visual elements, such as colours, imagery and displays, play a significant role in shaping consumer perceptions and strengthening brand identity (Bui and Nguyen, 2022). Brands like Apple and Tesla often use neutral colour palettes, such as white and grey, to convey sophistication, exclusivity and superior quality, thereby reinforcing their elite brand image (Derval, 2022). As traditional marketing strategies become oversaturated, businesses are increasingly relying on sensory cues as a neuromarketing strategy to cut through market noise and influence consumer behaviour on a subconscious level (Shahid et al., 2022). Research also supports aromatically enhanced environments, that enhance affective engagement and provides immediate gratification (Pandey and Tripathi, 2025). Furthermore, effectiveness of ambient music in retail settings (Garlin and Owen, 2006), quicker movement associated with upbeat and fast-paced music (Mohan et al., 2013). Nonetheless, there is a dearth of neuromarketing studies that connect sensory triggers to consumer impulsivity across a range of consumer traits. Therefore, we propose the following hypothesis to explore this intersection:

*H3*: Sensory triggers significantly impact neuromarketing efficacy in advertisement.

#### 2.2.4 Neuro-pricing strategies

Neuro-pricing strategies refer to psychologically informed pricing techniques that shape value perception through cognitive heuristics (Boz et al., 2017). Techniques such as charm pricing, anchoring, and reference price framing influence perceived affordability and attractiveness while minimizing extensive deliberative processing. These strategies go beyond pricing decisions, in other words, it makes the brain sway, bypassing logical processing in a scientifically substantiated manner (Oliveira et al., 2022). Marketers tactfully deploy practices such as anchoring, where they present a higher price first to make subsequent lower prices appear more reasonable, or charm pricing, i.e., priced at $9.99 instead of a round figure like $10, to influence consumer emotions, capture attention and shape judgments (Alsharif et al., 2023). Lee et al. (2007) asserts that such strategies are instrumental in steer favourable consumer perceptions, ultimately leading to higher profit margins. Additionally, perceived pricing signals are crucial in value creation and satisfaction levels, highlighting the impact of neuromarketing effectiveness (Garczarek-Bąk et al., 2021). Although neuro-pricing strategies have attracted scholarly attention, there is still a notable gap in the literature concerning consumer impulsivity and role of individual attributes. Consequently, this paper aims to investigate the interaction between these variables and presents the following hypothesis:

*H4*: Neuropricing strategies significantly impact neuromarketing efficacy in advertisement.

#### 2.2.5 Endorsement influence

Endorsement influence refers to the persuasive effect generated when a notable public figure, influencer, or satisfied customer publicly advocates a brand (Bergkvist and Zhou, 2016). Endorsements may enhance trust, familiarity, and identification, thereby increasing perceived marketing credibility and effectiveness (Zhu et al., 2022). This advocacy not only reinforces the trust and elevates the perceived value but also creates a sense of social validation that decisively influences consumer attitudes and purchasing decisions (Knoll and Matthes, 2017). Within a competitive landscape, it aids in distinguishing the market presence, elevating brand reputation and potentially driving increased sales. Nike’s Air Jordan demonstrates the significant impact of this strategy, as evidenced by the remarkable surge in sales following endorsement by Michael Jordan (Atkinson and DeWitt, 2016). Neuromarketing studies have observed this phenomenon by monitoring neural responses through eye-tracking patterns (Bergkvist and Zhou, 2016) and purchase intentions among socially influenced individuals (Pop et al., 2021). Similarly, other methods such as heart rate monitoring and skin conductance have been utilized in neuroscientific studies to assess the impact of endorsements on consumer purchasing behaviour (Baldo et al., 2022). Furthermore, the advent of live streaming advertisements on social media platforms has proven to be a powerful catalyst for impulsive buying, compelling consumers to make spontaneous purchasing decisions (Pop et al., 2021). Nonetheless, there exists a void in the literature to explore how endorsements influence immediate buying behaviour, contextual to neurological responses, particularly among consumers with specific traits. To address this gap, the following hypothesis is proposed:

*H5*: Endorsement influences significantly impact neuromarketing efficacy in advertisement.

#### 2.2.6 Cognitive processing cues

Cognitive processing involves conscious and rational functions of the brain that interprets, evaluate and respond to advertising stimuli (Adalarasu et al., 2025). By enhancing clarity, transparency, and evaluative confidence, such cues may strengthen perceived neuromarketing efficacy through reinforced credibility rather than purely affective activation. Research by Zito et al. (2021) suggests that when marketing messages are aligned with consumer’s inherent processing tendencies, it amplifies receptivity, emotional resonance and elicits favourable responses. As persuasive marketing continually evolving into subtle influences, there is a notable shift from social paradigms to scientifically grounded approaches, rooted in cognitive processing (Casado-Aranda et al., 2023). According to Zafar et al. (2020), strategies that effectively engage consumer’s fast-processing systems stimulate high impulsivity, especially when paired with emotionally charged or sensory cues. Usage of diverse neuro-imaging tools has given researchers the deep understanding of unprecedented consumer behaviour and mental dispositions that conventional marketing often fails to embrace (Alsharif et al., 2023). However, it is crucial to note, that not all consumers react impulsively to neuromarketing stimuli. Some studies suggest that high cognitive engagement can potentially reduce the influence of neuromarketing by counteracting emotional tactics (Sharma et al., 2010), while others contend that even rational consumers exhibit neural susceptibility to well-designed stimuli (Ahmed et al., 2022). This inconsistency necessitates the study of individual-specific traits mediating the relationship between neuromarketing efficacy and impulse buying. Guided by this framework, this study presents the following hypothesis:

*H6*: Cognitive processing significantly impact neuromarketing efficacy in advisements.

#### 2.2.7 Neuromarketing efficacy and consumer impulsivity

Within the S–O–R framework, neuromarketing efficacy functions as the organismic transmission mechanism linking stimulus exposure to behavioural response. It captures the extent to which consumers perceive marketing stimuli as psychologically compelling and influential (Khondakar et al., 2024). When marketing stimuli are evaluated as effective, the likelihood of impulsive purchasing increases (Goncalves et al., 2024). Consumer impulsive behaviour is defined in this study as spontaneous and unplanned purchasing behaviour triggered by immediate psychological activation (Floh and Madlberger, 2013). Accordingly, higher perceived neuromarketing efficacy is expected increase consumer impulsive behaviour. Based on reviewed literature, study presents the following hypothesis:

*H7*: Neuromarketing efficacy significantly impacts consumer impulsivity.

#### 2.2.8 Moderating role of consumer traits

Impulsive behaviour is not solely driven by external stimuli; but also shaped by enduring dispositional tendencies (Zito et al., 2021). Consumer traits reflect relatively stable psychological predispositions that influence how individuals process persuasive information and regulate their affective responses. Individuals high in emotional susceptibility, FOMO (Hodkinson, 2019), and sensitivity to social influence are more likely to rely on intuitive processing and respond more strongly to affect-laden marketing cues.

Digital environments intensify these tendencies through mechanisms such as social proof, influencer engagement, and personalized promotions (Kang et al., 2020). From a dual-process perspective, such individuals are more inclined towards intuitive processing pathways, increasing their likelihood of translating affective arousal into impulsive purchasing behaviour. Accordingly, consumer traits may directly influence consumer impulsivity.

Apart from their direct effects, consumer traits may also function as boundary conditions within the S–O–R framework. Even when neuromarketing stimuli are perceived as effective, the strength translation into impulsive behaviour depends on an individual’s dispositional susceptibility (Rodrigues et al., 2021). Consumers with higher levels of emotional and social sensitivities are expected to exhibit stronger responses to perceived neuromarketing efficacy. Based on above literature following hypotheses were derived:

*H8*: Consumer traits significantly impact the consumer impulsivity.

*H9*: Consumer traits moderate the relationship between neuromarketing efficacy and consumer impulsivity.

## 3 Materials and methods

### 3.1 Research design

This study employed a quantitative, cross-sectional research design to examine the effects of neuromarketing determinants on consumer impulsive behaviour, while accounting for the moderating influence of consumer traits. A cross-sectional design is appropriate for testing theoretically grounded structural relationships and boundary conditions within behavioural research contexts. The design enables simultaneous examination of direct, mediating, and moderating effects within an integrated structural model.

### 3.2 Instrument development

The survey instrument was developed based on established theoretical frameworks and previously validated measurement scales. Items were adapted from prior empirical studies (Smidts et al., 2014; Plassmann et al., 2015; Nguyen and Dang, 2022; Alsharif et al., 2023; Kala et al., 2023; Behl et al., 2024). All constructs were operationalised using multi-item reflective measures and assessed on a five-point Likert scale (1 = strongly disagree to 5 = strongly agree). To ensure conceptual and contextual alignment, items were reviewed for consistency with the S–O–R framework and adapted to reflect digitally mediated neuromarketing environments. Content validity was established through theoretical alignment and expert review. Minor linguistic modifications were implemented to improve clarity and contextual relevance without altering the underlying conceptual meaning. The adaptation process preserved construct integrity while ensuring applicability to the target population.

### 3.3 Pilot testing

A pilot study (*n* = 50) was conducted in the Delhi–NCR region to evaluate instrument clarity, reliability, and preliminary construct validity. Reliability assessment indicated that Cronbach’s alpha values for all constructs exceeded the recommended threshold of 0.70 (Nunnally and Bernstein, 1994). Composite reliability values met or exceeded 0.70, and average variance extracted (AVE) values surpassed the 0.50 criterion, confirming convergent validity. Indicator loadings exceeded 0.60, demonstrating acceptable item reliability. Feedback from pilot participants led to minor wording refinements to enhance comprehension and response accuracy. No structural modifications to the measurement model were required prior to full-scale data collection.

### 3.4 Sampling procedure and bias assessment

The target population comprised consumers residing in the Delhi–NCR region, frequently engaged in internet-based activities, including social networking, online shopping, entertainment consumption, and information search, and who are regularly exposed to digital advertising and neuromarketing-related stimuli.

A non-probability convenience sampling approach was employed due to accessibility constraints and the exploratory nature of the study. While convenience sampling may limit probabilistic generalizability, several procedural safeguards were implemented to enhance sampling rigor. First, screening questions ensured that respondents had prior exposure to neuromarketing-driven advertisements. Second, offline self-administered questionnaires were used to broaden participation beyond purely online-accessible populations. Third, demographic characteristics were monitored during data collection to reduce over-representation of specific subgroups.

A total of 629 questionnaires were distributed. Following data screening procedures 20 responses were excluded, including checks for missing values, multivariate outliers, inconsistent response patterns, and failed attention checks,. The final dataset comprised 609 valid responses (effective response rate ≈ 97%). This sample size exceeds recommended minimum thresholds for structural equation modelling and is adequate for PLS-SEM analyses involving mediation and moderation effects (Kline, 2023).

To assess potential non-response bias, early and late respondents were compared across key constructs using independent sample tests. No statistically significant differences were observed, suggesting that non-response bias is unlikely to materially affect the findings.

### 3.5 Common method bias (CMB) control

Given the cross-sectional design and reliance on self-reported data, procedural and statistical remedies were implemented to mitigate potential common method bias (Podsakoff et al., 2003). Procedurally, respondents were assured of anonymity and confidentiality to reduce evaluation apprehension and social desirability bias. Predictor and criterion variables were psychologically separated within the questionnaire to minimize respondent’s ability to infer hypothesized relationships. Item order was randomized, and attention-check questions were included to identify inattentive responses.

Statistically, Harman’s single-factor test was conducted to determine whether a single latent factor accounted for the majority of covariance among the measures. The results indicated that no single factor explained a dominant proportion of variance. In addition, full collinearity variance inflation factor (VIF) values were below the conservative threshold of 3.3. Collectively, these findings suggest that common method bias does not pose a significant threat to the validity of the results.

### 3.6 Data analysis strategy

Data were screened, coded, and cleaned using SPSS 22.0 prior to structural modelling. Hypothesized relationships were tested using Partial Least Squares Structural Equation Modelling (PLS-SEM) with SmartPLS 4.0.

The analysis followed a two-step approach. First, the measurement model was evaluated by assessing indicator reliability, internal consistency reliability (Cronbach’s alpha and composite reliability), convergent validity (AVE), and discriminant validity using the Fornell–Larcker criterion and the Heterotrait-Monotrait ratio (HTMT). All HTMT values remained below the recommended threshold of 0.90, indicating satisfactory discriminant validity. Near-threshold HTMT values were examined and interpreted as reflecting conceptual relatedness among neuromarketing determinants rather than construct redundancy.

Second, the structural model was assessed by examining path coefficients, bootstrapped t-values (5,000 resample), and significance levels. VIF values for predictor constructs were below critical thresholds, indicating no multicollinearity concerns. Moderation effects were tested using interaction term modelling. Interaction VIF values confirmed the absence of multicollinearity between main and interaction effects. Simple slope analysis and graphical interaction plots were generated to interpret the nature of the moderating relationship.

## 4 Result and findings

### 4.1 Demographic characteristics

Table 1 presents the demographic profile of the respondents (*N* = 609). With respect to age, the largest proportion of respondents belong to 35–45 years category (217; 35.6%), followed by those aged 45–55 years (155; 25.5%). Participants aged 25–35 years constitute 110 respondents (18.1%), while 105 respondents (17.2%) are up to 25 years old. Only a small segment is above 55 years (22; 3.6%), indicating that the sample is predominantly composed of middle-aged individuals. In terms of gender, males represent a slightly higher proportion (335; 55.0%) compared to females (274; 45.0%). Regarding marital status, the majority of respondents are married (459; 75.4%), whereas 112 (18.4%) are unmarried and 38 (6.2%) are separated or divorced. Educational qualification shows that most respondents possess postgraduate degrees (229; 37.6%), followed by graduation-level education (138; 22.7%) and intermediate education (94; 15.4%). Technical diplomas or certificates account for 74 respondents (12.2%), while 46 (7.6%) have education up to Matric level and 28 (4.6%) hold professional qualifications or other credentials. Concerning occupation, businesspersons and service employees each constitute 164 respondents (26.9% each), forming the largest occupational groups. Students account for 115 (18.9%), professionals for 80 (13.1%), housewives for 76 (12.5%), and others for 10 respondents (1.6%). Overall, the sample reflects a predominantly married, middle-aged, and well-educated population with diverse occupational representation.

**Table 1:** Demographic characteristics.

Characteristics	Description	Frequency	Percent
Age	Up to 25 years	105	17.2
	25–35 years	110	18.1
	35–45 years	217	35.6
	45 to 55 years	155	25.5
	Above 55 years	22	3.6
Gender	Male	335	55.0
	Female	274	45.0
Marital status	Unmarried	112	18.4
	Married	459	75.4
	Separated/divorcee	38	6.2
Education qualification	Up to matric level	46	7.6
	Up to intermediated	94	15.4
	Up to graduation	138	22.7
	Post-graduation	229	37.6
	Technical degree/diploma certificates	74	12.2
	Professional qualifications and others	28	4.6
Occupation	Student	115	18.9
	Business	164	26.9
	Service	164	26.9
	Housewives	76	12.5
	Professionals	80	13.1
	Others	10	1.6

Table 2 presents the descriptive statistics and factor loadings for constructs related to neuromarketing stimuli, consumer traits, and consumer impulsivity based on a sample of 609 respondents. Overall, all measurement items demonstrate strong factor loadings (ranging from 0.678 to 0.949), exceeding the recommended threshold of 0.60, thereby confirming adequate indicator reliability and internal consistency across constructs. Among the neuromarketing stimulus factors, Sensory Triggers reported the highest mean (M = 4.0928, SD = 0.73380, Var = 0.538), indicating that attractive packaging, pleasant music, ambient scents, and visually appealing displays strongly influence impulse buying behaviour. This is followed by Scarcity and Urgency (M = 3.6330, SD = 0.99100, Var = 0.982) and Emotional Appeals (M = 3.5074, SD = 0.84897, Var = 0.721), suggesting that FOMO cues, limited-time offers, and emotionally charged advertisements moderately to strongly stimulate impulsive purchases. Cognitive Processing also shows a moderate mean (M = 3.4486, SD = 0.73171, Var = 0.535), implying that eye-catching visuals, storytelling, and persuasive cues enhance brand recall and quicker decision-making. In contrast, Endorsement Influence (M = 3.3190, SD = 0.86327, Var = 0.745) and Neuro Pricing Strategies (M = 3.2422, SD = 0.97493, Var = 0.950) display relatively lower but still moderate mean scores, indicating comparatively weaker yet meaningful effects of social proof and psychological pricing on impulsive buying. Furthermore, Neuromarketing Efficacy demonstrates a moderate-to-high mean (M = 3.4864, SD = 0.61438, Var = 0.377), reflecting positive attitudes towards gamification and its motivational influence. Consumer Traits exhibit a relatively high mean (M = 3.8448, SD = 0.69915, Var = 0.489), suggesting that respondents are highly susceptible to targeted marketing, social media influence, and mood-driven shopping tendencies. Finally, Consumer Impulsivity shows a moderately high mean (M = 3.6483, SD = 0.69372, Var = 0.481), indicating that emotional triggers, strategic product placement, and subconscious brand recognition significantly contribute to unplanned purchasing behaviour. Collectively, the moderate standard deviations and variances across constructs reflect acceptable dispersion, indicating reasonable agreement among respondents while maintaining sufficient variability for further structural analysis.

**Table 2:** Descriptive statistics.

Code	Construct	Factor	Mean	SD	Var
*EA*	*Emotional appeals*		3.5074	0.84897	0.721
EA1	Advertisements that evoke strong emotions (happiness, excitement) make me purchase products impulsively.	0.857	3.3465	1.07291	1.151
EA2	Seeing emotionally engaging advertisements increases my likelihood of making unplanned purchases.	0.870	3.2906	1.01596	1.032
EA3	Fear-of-missing-out (FOMO) in advertisements makes me purchase immediately without much thought.	0.886	3.5665	0.92441	0.855
EA4	Ads featuring emotionally charged content (joy, urgency) significantly influence my buying decisions.	0.828	3.8259	0.93326	0.871
*SU*	*Scarcity and urgency*		3.6330	0.99100	0.982
SU1	Limited-time offers encourage me to buy products impulsively.	0.909	3.6420	1.09271	1.194
SU2	I feel an urge to purchase when I see “only a few items left” messages.	0.879	3.4910	1.17555	1.382
SU3	Flash sales and countdown timers push me to buy products even when I do not initially intend to.	0.891	3.5878	1.06958	1.144
SU4	I often buy products immediately when I see phrases like “Limited Stock Available” or “Hurry, Offer Ends Soon!.”	0.939	3.8112	1.03498	1.071
*ST*	*Sensory triggers*		4.0928	0.73380	0.538
ST1	Attractive packaging strongly influences my decision to purchase a product impulsively.	0.826	3.9622	1.05921	1.122
ST2	Pleasant background music in retail stores enhances my likelihood of making impulse purchases.	0.857	4.3448	0.65593	0.430
ST3	Stores with inviting scents and ambient settings make me more likely to buy products impulsively.	0.678	4.1166	0.83580	0.699
ST4	Visually appealing product displays grab my attention and lead me to unplanned purchases.	0.924	3.9475	0.89985	0.810
*NPS*	*Neuro pricing strategies*		3.2422	0.97493	0.950
NPS1	I am more likely to buy a product priced psychologically.	0.732	3.3793	1.23103	1.515
NPS2	Discounts and personalized pricing make me feel like I am getting a great deal, leading to impulsive purchases.	0.736	3.2217	1.28572	1.653
NPS3	Seeing a product’s price slashed from a higher amount (e.g., $199 Now $149) encourages me to buy immediately.	0.751	3.2053	1.31623	1.732
NPS4	Dynamic pricing (changing prices based on demand) influences my purchasing decisions.	0.804	3.1626	1.30088	1.692
*EI*	*Endorsement influence*		3.3190	0.86327	0.745
EI1	Positive online reviews make me purchase a product without much deliberation.	0.831	3.6092	1.03951	1.081
EI2	Recommendations from social media influencers significantly influence my buying decisions.	0.835	3.6059	1.03031	1.062
EI3	Seeing my peer’s purchase a product makes me feel compelled to buy it as well.	0.746	3.2282	1.15513	1.334
EI4	I am more likely to make an impulsive purchase when I see many people endorsing a product.	0.768	2.8325	1.12749	1.271
*CP*	*Cognitive process*		3.4486	0.73171	0.535
CP1	I tend to focus more on advertisements that use eye-catching visuals and sound effects.	0.923	3.4401	0.85845	0.737
CP2	Advertisements that use storytelling and emotional appeal influence my purchasing decisions.	0.902	3.3793	0.76238	0.581
CP3	I can recall brands more easily when they use unique sensory marketing techniques (e.g., music, colours, or scents).	0.927	3.6125	0.78487	0.616
CP4	I often feel inclined to purchase a product without realizing how the advertisement influenced me.	0.924	3.6026	0.76717	0.589
CP5	I make quicker purchasing decisions when advertisements use persuasive neuromarketing techniques, such as scarcity or urgency cues.	0.905	3.2085	0.82569	0.682
*NME*	*Neuromarketing efficacy*		3.4864	0.61438	0.377
NME1	I have a pleasant feeling towards gamification	0.949	3.5993	0.69802	0.487
NME2	I am very much enthusiast in gamification that adds value to the service/product.	0.816	3.2135	0.87945	0.773
NME3	Using gamified services/products is a positive experience.	0.936	3.5550	0.57982	0.336
NME4	Gamification motivates me to use the service/product more frequently.	0.932	3.5337	0.58146	0.338
NME5	I believe gamification is an effective tool for learning and improving skills.	0.946	3.5304	0.64084	0.411
*CT*	*Consumer traits*		3.8448	0.69915	0.489
CT1	Missing a good deal makes me anxious, and I shop to boost my mood.	0.844	3.7668	0.85541	0.732
CT2	I like discovering new products, influenced by ads and influencers.	0.904	3.7882	0.85231	0.726
CT3	Social media shapes my shopping habits through ads and promotions.	0.798	3.8473	0.74427	0.554
CT4	Targeted marketing makes impulse buying hard to resist.	0.792	3.7767	0.89922	0.809
CT5	Online marketing and personalized ads have increased my impulsive buying.	0.911	3.9458	0.69445	0.482
CT6	I am more likely to make impulsive purchases when advertisements use appealing colours, sounds, or scents.	0.937	3.9442	0.81526	0.665
*CI*	*Consumer impulsivity*		3.6483	0.69372	0.481
CI1	I tend to buy products on impulse when advertisements evoke strong emotions like excitement or nostalgia.	0.903	3.7586	0.77110	0.595
CI2	I am more likely to make unplanned purchases when products are strategically placed in stores or online.	0.833	3.8144	0.86606	0.750
CI3	I often feel an urge to buy when brands use psychological triggers like scarcity (limited stock) or urgency (limited-time offers).	0.835	3.4466	0.90550	0.820
CI4	I sometimes make purchases without realizing how much advertisements or branding have influenced my decision.	0.773	3.7685	0.72361	0.524
CI5	I am more likely to buy impulsively when I see a brand that I subconsciously recognize and trust.	0.884	3.5205	0.83508	0.697
CI6	I often buy impulsively, especially with discounts.	0.949	3.4864	0.61438	0.377

### 4.2 Measurement model

Table 3 demonstrates satisfactory construct reliability and convergent validity for all latent variables in the measurement model. Internal consistency reliability is confirmed as Cronbach’s alpha values range from 0.756 to 0.952, exceeding the recommended threshold of 0.70 (acceptable) and remaining below the upper cautionary limit of 0.95 in most cases, thereby indicating strong reliability without serious redundancy concerns. Similarly, composite reliability estimates, both rho_a and rho_c range from 0.782 to 0.963, surpassing the recommended minimum value of 0.70 and confirming adequate construct reliability. Convergent validity is supported by AVE values between 0.572 and 0.841, all above the threshold of 0.50, indicating that each construct explains more than 50% of the variance of its indicators. Multicollinearity diagnostics show VIF values ranging from 1.005 to 1.999, well below the conservative cut-off value of 3.3 (and the maximum threshold of 5), suggesting the absence of multicollinearity issues. Furthermore, the f-square effect sizes indicate varying magnitudes of structural impact, with Neuromarketing Efficacy (0.836) demonstrating a large effect (≥0.35), Consumer Impulsivity (0.454) and Emotional Appeals (0.415) showing substantial effects, Cognitive Process (0.236) and Sensory Triggers (0.098) indicating moderate to small effects (0.02–0.15), and Consumer Traits (0.022), Endorsement Influence (0.005), Scarcity and Urgency (0.006), and Neuro Pricing Strategies (0.059) reflecting small to negligible effects. Overall, the measurement model exhibits strong reliability, adequate convergent validity, and no multicollinearity concerns.

**Table 3:** Construct reliability and validity.

Constructs	Cronbach’s alpha	rho_a	rho_c	AVE	VIF	f-square
Cognitive process	0.952	0.954	0.963	0.839	1.999	0.236
Consumer impulsivity	0.916	0.921	0.934	0.704	1.306	0.454
Consumer traits	0.932	0.933	0.947	0.750	1.010	0.022
Emotional appeals	0.883	0.888	0.919	0.741	1.750	0.415
Endorsement influence	0.806	0.807	0.873	0.633	1.393	0.005
Neuromarketing efficacy	0.952	0.955	0.963	0.841	1.296	0.836
Neuro pricing strategies	0.756	0.782	0.842	0.572	1.011	0.059
Scarcity and urgency	0.928	0.974	0.947	0.818	1.005	0.006
Sensory triggers	0.860	0.964	0.895	0.682	1.006	0.098

Table 4 presents the combined discriminant validity results using the Fornell–Larcker criterion and the HTMT ratio. The diagonal elements represent the square root of AVE, and all constructs demonstrate adequate convergent validity, as the square root of AVE values: Cognitive Process (0.916), Consumer Impulsivity (0.839), Consumer Traits (0.866), Emotional Appeals (0.861), Endorsement Influence (0.796), Neuromarketing Efficacy (0.917), Neuro Pricing Strategies (0.756), Scarcity and Urgency (0.905), and Sensory Triggers (0.826) are greater than their corresponding inter-construct correlations in the lower triangular matrix. This confirms that each construct shares more variance with its own indicators than with other constructs, thereby satisfying the Fornell–Larcker criterion for discriminant validity. Furthermore, the HTMT values reported in the upper triangular matrix are all below the conservative threshold of 0.90 (the highest being 0.875 between Cognitive Process and Consumer Impulsivity, and 0.830 between Consumer Impulsivity and Emotional Appeals), indicating that discriminant validity is statistically established at the recommended level. Although some construct pairs, including Cognitive Process–Consumer Impulsivity and Emotional Appeals–Neuromarketing Efficacy reveal relatively higher associations, they remain within acceptable limits and do not threaten discriminant validity. Therefore, the measurement model demonstrates adequate discriminant validity, confirming that all constructs are empirically distinct and statistically reliable for subsequent structural model analysis.

**Table 4:** Combined discriminant validity.

Construct	CP	CI	CT	EA	EI	NME	NPS	SU	ST
CP	**0.916**	0.875	0.567	0.704	0.586	0.736	0.042	0.034	0.051
CI	0.828	**0.839**	0.731	0.830	0.616	0.786	0.051	0.078	0.092
CT	0.536	0.677	**0.866**	0.539	0.403	0.498	0.086	0.082	0.135
EA	0.649	0.760	0.485	**0.861**	0.472	0.798	0.056	0.044	0.094
EI	0.516	0.530	0.350	0.401	**0.796**	0.467	0.092	0.063	0.047
NME	0.713	0.757	0.478	0.744	0.416	**0.917**	0.187	0.078	0.204
NPS	0.014	−0.015	0.041	0.030	−0.063	0.157	**0.756**	0.054	0.089
SU	0.024	0.069	0.074	0.029	0.045	0.078	0.045	**0.905**	0.058
ST	0.029	0.021	−0.097	0.040	−0.029	0.207	0.029	0.058	**0.889**

Bold values provided to CP = Cognitive Process, CI = Consumer Impulsivity, CT = Consumer Traits, EA = Emotional Appeals, EI = Endorsement Influence, NME = Neuromarketing Efficacy, NPS = Neuro Pricing Strategies, SU = Scarcity and Urgency, ST = Sensory Triggers.

### 4.3 Structural model and hypothesis testing

Table 5 presents the R-square (*R*^2^), adjusted R-square, Q^2^predict, and global model fit indices, demonstrating satisfactory explanatory power, predictive relevance, and acceptable model fit. The structural model explains a substantial proportion of variance in the endogenous constructs, with *R*^2^ values of 0.708 for Consumer Impulsivity and 0.697 for Neuromarketing Efficacy, indicating that approximately 70.8 and 69.7% of the variance in these constructs is accounted for by the predictor variables. The adjusted *R*^2^ values (0.707 and 0.694, respectively) are nearly identical to the *R*^2^ values, suggesting model stability and minimal shrinkage. Furthermore, the Q^2^predict values of 0.689 (Consumer Impulsivity) and 0.748 (Neuromarketing Efficacy) are well above zero, confirming strong predictive relevance of the model. The relatively low RMSE (0.560 and 0.504) and MAE (0.460 and 0.392) values further support satisfactory predictive accuracy. Regarding overall model fit, the SRMR values for the saturated model (0.070) and estimated model (0.079) fall within the acceptable threshold of <0.08, indicating a good fit between the observed and predicted correlations. Although the NFI values (0.696 and 0.684) are slightly below the conventional 0.90 benchmark, they remain acceptable in variance-based SEM contexts. Additionally, the d_ULS and d_G values are lower in the saturated model compared to the estimated model, suggesting reasonable model consistency. Collectively, these statistics provide empirical justification for the adequacy, predictive capability, and overall fitness of the structural model.

**Table 5:** R-square, Q-square and model fit summary.

Constructs	R-square	R-square adjusted	Q^2^predict	RMSE	MAE
Consumer impulsivity	0.708	0.707	0.689	0.560	0.460
Neuromarketing efficacy	0.697	0.694	0.748	0.504	0.392

Model fit summary	Saturated model	Estimated model
SRMR	0.070	0.079
d_ULS	4.385	5.691
d_G	2.930	3.109
Chi-square	8576.596	8909.534
NFI	0.696	0.684

**Figure 2:**
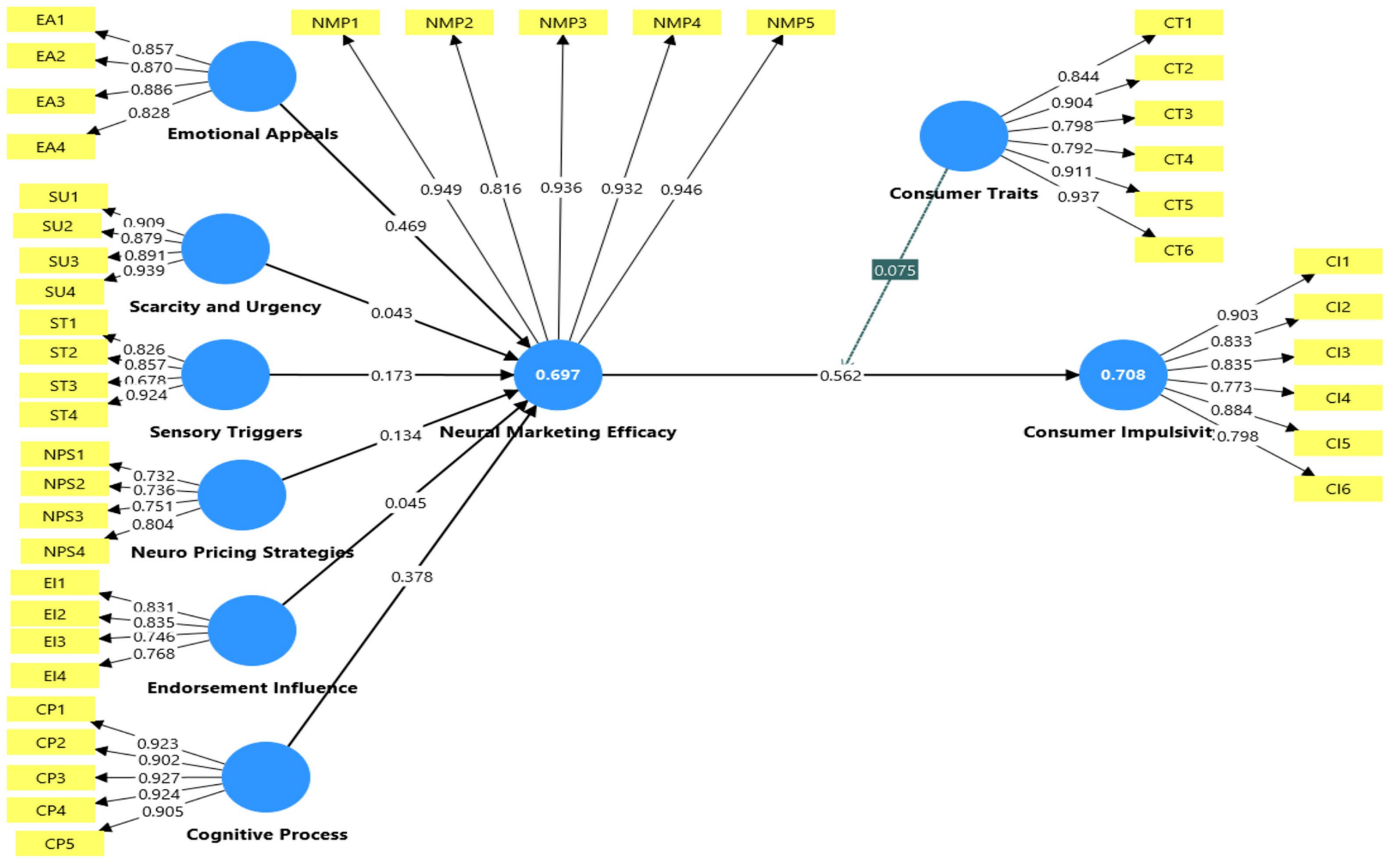
Structural model.

Table 6 and Figure 2 present the structural model outcomes. The structural model results indicate that Emotional Appeals (*β* = 0.469, *t* = 13.704, *p* < 0.001), Sensory Triggers (*β* = 0.173, *t* = 8.277, *p* < 0.001), Neuro-Pricing Strategies (*β* = 0.134, *t* = 5.418, *p* < 0.001), and Cognitive Processing (*β* = 0.378, *t* = 9.533, *p* < 0.001) exert a strong and statistically significant positive effect on Neuromarketing Efficacy. In contrast, Scarcity and Urgency cues (*β* = 0.043, *t* = 1.670, *p* = 0.095) and Endorsement Influence (*β* = 0.045, *t* = 1.524, *p* = 0.127) do not reach statistical significance at the 0.05 level. These findings indicate that, within the present sample, these determinants do not meaningfully contribute to perceived neuromarketing efficacy.

**Table 6:** Structural model outcome.

Structural relationship	Path coefficient (*β*)	Standard deviation (STDEV)	T statistics (|O/STDEV|)	*p-*values	Remarks
Emotional appeals → neuromarketing efficacy	0.469	0.034	13.704	0.000	Significant
Scarcity and urgency → neuromarketing efficacy	0.043	0.026	1.670	0.095	Insignificant
Sensory triggers → neuromarketing efficacy	0.173	0.021	8.277	0.000	Significant
Neuro pricing strategies → neuromarketing efficacy	0.134	0.025	5.418	0.000	Significant
Endorsement influence → neuromarketing efficacy	0.045	0.029	1.524	0.127	Insignificant
Cognitive process → neuromarketing efficacy	0.378	0.040	9.533	0.000	Significant
Neuromarketing efficacy → consumer impulsivity	0.562	0.026	21.249	0.000	Significant
Consumer traits → consumer impulsivity	0.416	0.030	13.956	0.000	Significant
Consumer traits × neuromarketing efficacy → consumer impulsivity	0.075	0.018	4.065	0.000	Significant

Neuromarketing Efficacy significantly predicts Consumer Impulsivity (*β* = 0.562, *t* = 21.249, *p* < 0.001), indicating a strong positive relationship between perceived marketing effectiveness and spontaneous purchasing behaviour. Consumer Traits also exert a significant direct effect on Consumer Impulsivity (*β* = 0.416, *t* = 13.956, *p* < 0.001). The interaction term between Consumer Traits and Neuromarketing Efficacy is statistically significant (*β* = 0.075, *t* = 4.065, *p* < 0.001), confirming the proposed moderating effect. This result indicates that the positive relationship between Neuromarketing Efficacy and Consumer Impulsivity becomes stronger at higher levels of dispositional susceptibility.

Overall, the structural model demonstrates that most hypothesized relationships are supported, with the exception of Scarcity and Urgency cues and Endorsement Influence. The result patterns suggests that affective and cognitively reinforced stimuli play a more substantial role in shaping perceived neuromarketing effectiveness than scarcity-based or endorsement-based cues within this context.

### 4.4 Moderation analysis

**Figure 3:**
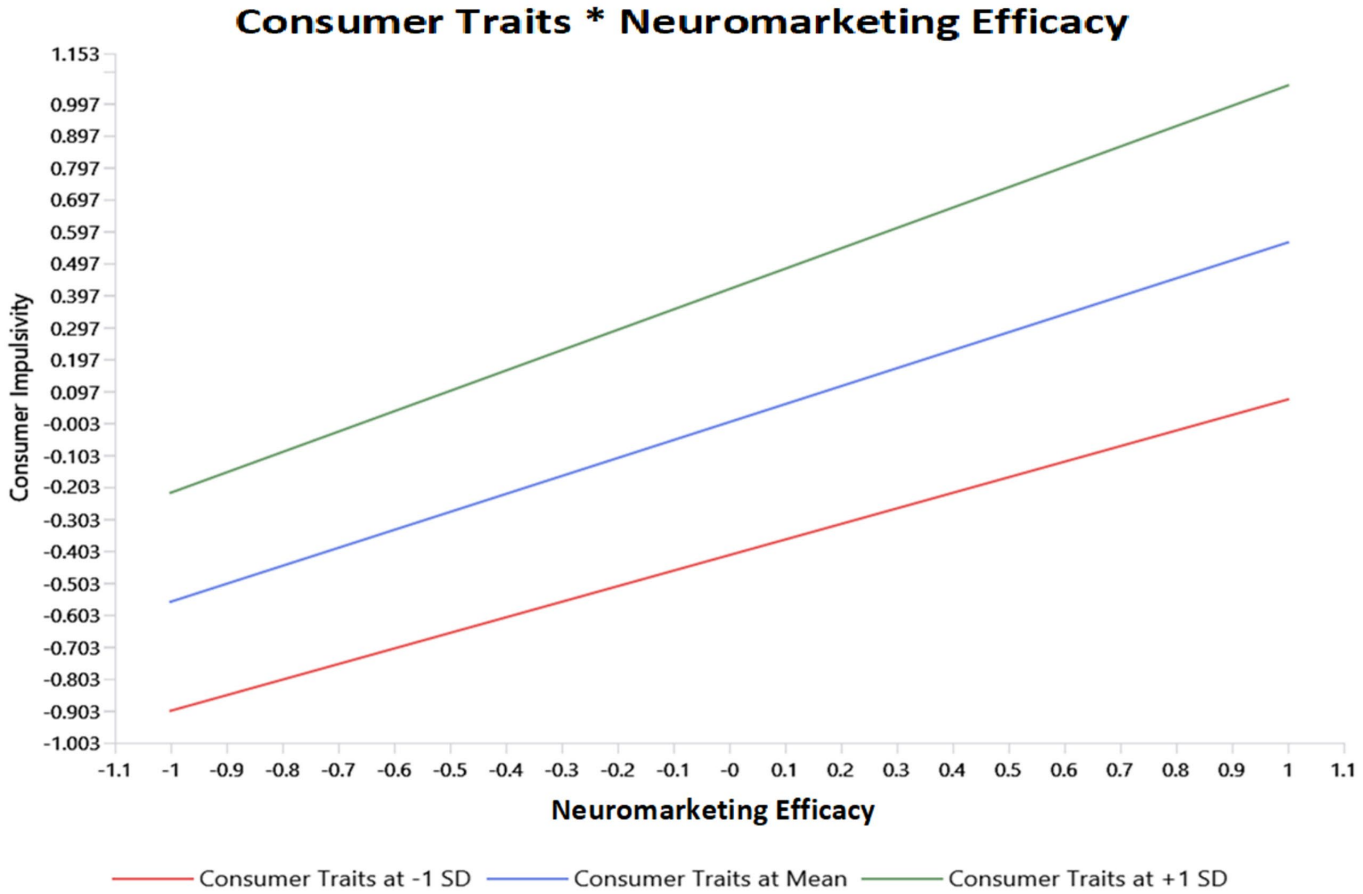
Slope analysis.

The moderation analysis presented in Table 6 and Figure 3 examines the structural relationships influencing consumer impulsivity, particularly the moderating effect of Neuromarketing Efficacy on the relationship between Consumer Traits and Consumer Impulsivity. Furthermore, Neuromarketing Efficacy significantly predicts Consumer Impulsivity (*β* = 0.562, *t* = 21.249, *p* < 0.001), indicating a strong direct effect. Consumer Traits also significantly influence Consumer Impulsivity (*β* = 0.416, *t* = 13.956, *p* < 0.001). Finally, the interaction term between Consumer Traits and Neuromarketing Efficacy (*β* = 0.075, *t* = 4.065, *p* < 0.001) is significant, confirming the moderating effect and supporting the moderation hypothesis. Overall, the structural model demonstrates that most hypothesized paths are statistically significant, with the exception of Scarcity & Urgency and Endorsement Influence. The statistical significance of all relationships, as indicated by *p*-values below 0.05 and high t-values exceeding 1.96, supports the robustness of these findings. These results imply that consumer traits amplify the influence of Neuromarketing Efficacy on Consumer Impulsivity, reinforcing the critical role of marketing strategies that leverage neuroscientific insights to drive consumer behaviour.

## 5 Discussion

This study examined how six neuromarketing determinants influence consumer impulsive behaviour through perceived neuromarketing efficacy, while accounting for the moderating role of consumer traits within an S–O–R framework. The findings provide insight into the relative strength of affective, cognitive, and dispositional mechanisms underlying impulsive consumption in digitally mediated contexts.

Among the determinants, Emotional Appeals emerged as the strongest predictor of Neuromarketing Efficacy (*β* = 0.469, *p* < 0.001). This result supports affect-dominant decision models, indicating that emotionally salient stimuli enhance perceived persuasive effectiveness and facilitate rapid evaluative processing. Emotional activation appears to reduce cognitive resistance and increase readiness for spontaneous purchasing, consistent with System 1 processing dynamics.

Cognitive Processing also demonstrated a strong positive effect (*β* = 0.378, *p* < 0.001). Rather than contradicting impulsivity frameworks, this finding suggests that structured informational cues enhance evaluative confidence and apprehended credibility, thereby strengthening perceived marketing effectiveness. In this context, cognitive reinforcement may amplify the organismic evaluation (neuromarketing efficacy), which subsequently translates into impulsive action. This result indicates that impulsive behaviour can be influenced by both affective activation and cognitively reinforced persuasion.

Sensory Triggers (*β* = 0.173, *p* < 0.001) and Neuro-Pricing Strategies (*β* = 0.134, *p* < 0.001) also significantly enhanced neuromarketing efficacy. These results align with sensory marketing and pricing heuristic theories, suggesting that multisensory engagement and value framing operate through heuristic evaluation processes rather than extensive analytical deliberation. Although their effect sizes are smaller relative to emotional and cognitive cues, they remain meaningful contributors to perceived marketing effectiveness.

In contrast, Scarcity and Urgency cues (*β* = 0.043, *p* = 0.095) and Endorsement Influence (*β* = 0.045, *p* = 0.127) did not demonstrate significant effects. While prior literature frequently highlights scarcity-based and endorsement mechanisms as impulse triggers, the present findings suggest diminished incremental influence within digitally saturated environments. Repetitive exposure to time-bound promotions and influencer endorsements may attenuate their persuasive impact, potentially due to reduced perceived authenticity.

Consistent with the hypothesized mediation, Neuromarketing Efficacy significantly predicted Consumer Impulsivity (*β* = 0.562, *p* < 0.001), confirming its role as the psychological transmission mechanism linking marketing stimuli to behavioural response within the S–O–R model.

Consumer Traits exerted a notable effect on Impulsivity (*β* = 0.416, *p* < 0.001), underscoring the dispositional foundation of spontaneous purchasing behaviour. More importantly, the interaction between Consumer Traits and Neuromarketing Efficacy was significant (*β* = 0.075, *p* < 0.001), indicating that the relationship between perceived marketing effectiveness and impulsive behaviour strengthens at higher levels of dispositional susceptibility. This moderation effect confirms that neuromarketing responsiveness is conditioned by stable psychological tendencies rather than operating uniformly across consumers.

Taken together, the findings suggest an integrated mechanism in which affective and cognitively reinforced stimuli enhance perceived neuromarketing efficacy, which subsequently drives impulsive purchasing behaviour. This process is further amplified among consumers characterized by higher emotional susceptibility and social influence sensitivity, reinforcing the importance of trait-based boundary conditions in understanding neuromarketing effectiveness.

## 6 Study implications

### 6.1 Theoretical implications

This research contributes to neuromarketing and consumer behaviour literature by clarifying the psychological processes through which marketing stimuli translates into impulsive purchasing behaviour. First, the findings extend dual-process perspectives by demonstrating that both affect-driven and cognitively reinforced mechanisms shape impulsive responses. Consistent with System 1 processing frameworks, emotionally stimulating content, sensory cues, and heuristic-based pricing strategies significantly enhanced neuromarketing efficacy. Simultaneously, the significant effect of cognitive processing indicates that structured and credibility-enhancing information can reinforce evaluative confidence. Thus, impulsive behaviour in neuromarketing contexts appears to emerge from an interaction between intuitive activation and cognitively reinforced persuasion rather than purely affective arousal.

Second, the study extends the S–O–R framework (Mehrabian and Russell, 1974) by empirically validating neuromarketing efficacy as the mediating organismic mechanism linking marketing stimuli to impulsive responses. This clarifies the psychological transmission pathway between external cues and behavioural outcomes.

Third, the findings offer a nuanced perspective on the Theory of Planned Behaviour (Ajzen, 1991). While the theory emphasizes intention-driven action, the strong effect of neuromarketing efficacy on impulsivity suggests that in digitally intensive environments, automatic functions may function alongside or even supersede deliberate intentions.

Fourth, by incorporating consumer traits as moderators, the study establishes dispositional boundary conditions within neuromarketing research. The significant interaction effect demonstrates that individual differences in personality systematically shape responsiveness to marketing stimuli. This trait-contingent perspective advances understanding of heterogeneity in impulsive behaviour.

Collectively, these findings support the development of integrative models that incorporate affective, cognitive, and dispositional dimensions to more precisely capture contemporary consumer decision-making processes.

### 6.2 Managerial implications

The findings provide actionable guidance for marketers, advertisers, and brand strategists operating in digitally competitive environments. Given the strong effect of emotional appeals (*β* = 0.469), brands should prioritize affectively resonant storytelling that aligns with aspirational or identity-based themes. For example, short-form video campaigns on platforms such as Instagram Reels or YouTube Shorts may integrate emotionally engaging narratives rather than relying solely on product specifications.

The significant impact of cognitive processing (*β* = 0.378) suggests that emotion centric messaging should be complemented by structured and credibility-enhancing information. E-commerce interfaces may incorporate persuasive headlines alongside concise bullet-point value propositions, product certifications, or transparent comparison charts to reinforce evaluative confidence.

Since sensory triggers significantly enhanced perceived efficacy (*β* = 0.173), brands should refine digital design elements, including colour palettes, visual hierarchy, interface responsiveness, and auditory cues to enhance experiential immersion. Consistent multisensory branding across online and offline touch points may strengthen recall and engagement.

Given the effect of neuro-pricing strategies (*β* = 0.134), firms may implement psychologically informed pricing structures such as anchor comparisons, bundle framing, or charm pricing (e.g., ₹999 vs. ₹1,000) to activate heuristic-based value perception. However, these strategies should remain transparent to preserve consumer trust.

The significant moderating effect of consumer traits indicates that neuromarketing strategies should not be uniformly applied. Instead, organizations may adopt trait-informed segmentation approaches supported by AI-driven analytics. For example, novelty-seeking consumers may respond to product launch alerts, emotionally susceptible consumers to narrative-driven campaigns, and socially influenced consumers to peer reviews and social proof elements. Platforms such as Meta Ads Manager and CRM systems can support behavioural segmentation and creative optimization.

The non-significant effects of scarcity cues and endorsement influence suggest that excessive reliance on time-bound promotions or influencer partnerships may yield diminishing returns, underscoring the need for authenticity and contextual relevance.

Finally, the persuasive power of neuromarketing necessitates ethical responsibility. Overuse of emotionally manipulative or psychologically intrusive tactics may result in consumer fatigue or brand aversion (Yao and Wang, 2024). Sustainable competitive advantage depends on balancing persuasive effectiveness with transparency and long-term trust-building.

## 7 Conclusion

This study contributes to neuromarketing and consumer behaviour research by examining how perceived neuromarketing efficacy mediates the relationship between marketing stimuli and consumer impulsive behaviour, while consumer traits function as boundary conditions shaping responsiveness.

The findings suggest that emotionally driven content, cognitively structured messaging, sensory triggers, and neuro-pricing strategies are associated with higher levels of perceived neuromarketing efficacy, which in turn increases impulsive purchasing. Importantly, the moderation results confirm that these effects are stronger among dispositionally susceptible consumers, underscoring the trait-contingent nature of neuromarketing effectiveness.

By integrating stimulus characteristics, internal evaluative mechanisms, and dispositional heterogeneity within a unified structural framework, this research contributes to a more comprehensive understanding of impulsive consumption in digitally mediated environments.

Despite these contributions, several limitations warrant consideration. First, the study focused on digitally engaged consumers within the lifestyle product segment in the Delhi–NCR region of India. Generalization beyond similar urban and digitally saturated contexts should therefore be undertaken cautiously, as consumer responses may vary across product categories, income segments, and cultural environments.

Second, the cross-sectional design limits causal inference. Although structural relationships were statistically significant, longitudinal and experimental designs are necessary to examine temporal dynamics and causal mechanisms underlying neuromarketing effectiveness.

Third, while consumer traits were incorporated as moderating variables, the analysis emphasized foundational dispositional tendencies. Future research may extend the model by incorporating additional psychological constructs such as emotional intelligence, cognitive reflection, self-regulatory capacity, or behavioural inhibition to deepen understanding of impulse control mechanisms.

Fourth, the reliance on self-reported quantitative data may introduce perceptual bias despite procedural safeguards. As the present study relies on perception-based survey measures rather than direct neurophysiologic data, the findings should be interpreted as reflecting consumers’ perceived responses to neuromarketing-inspired marketing stimuli rather than neural activation itself. Integrating qualitative methods, such as interviews, focus groups, or behavioural observation may enrich interpretation of underlying motivational processes.

Fifth, cross-cultural replication studies are necessary to evaluate external validity across diverse socio-economic and cultural settings. Hence, such comparative analyses would strengthen the generalizability of the proposed framework.

Finally, as marketing ecosystems increasingly incorporate artificial intelligence and real-time personalisation systems, future research should investigate AI-driven neuromarketing practices. Examining how ethically designed AI systems interpret behavioural signals and deliver personalized stimuli may provide critical insight into evolving patterns of impulsive consumption in digitally intelligent environments.

In summary, neuromarketing effectiveness is shaped by affective, cognitive, and dispositional mechanisms and remains context-dependent. Continued interdisciplinary inquiry is essential to refine theoretical models and guide responsible marketing practices.

## Data Availability

The original contributions presented in the study are included in the article/supplementary material, further inquiries can be directed to the corresponding author.
